# Supplementary material for: Spin-Cherenkov effect in a magnetic nanostrip with interfacial Dzyaloshinskii-Moriya interaction
Source: Sci Rep. 2016 May 4;6:25189. doi: 10.1038/srep25189 (PMC4855169; doi:10.1038/srep25189)
Supplement: Supplementary Information [file srep25189-s1.pdf]

Supplementary Information for "Spin-Cherenkov effect in a magnetic nanostrip with interfacial Dzyaloshinskii-Moriya interaction"

Jing Xia,[1,2] Xichao Zhang,[1,2] Ming Yan,[3] Weisheng Zhao,[4,5,*] and Yan Zhou[1,2,6,†]

[1]*School of Electronics Science and Engineering, Nanjing University, Nanjing 210093, China*

[2]*Department of Physics, University of Hong Kong, Hong Kong, China*

[3]*Department of Physics, Shanghai University, Shanghai 200444, China*

[4]*Fert Beijing Institute, Beihang University, Beijing 100191, China*

[5]*School of Electronic and Information Engineering, Beihang University, Beijing 100191, China*

[6]*Center of Theoretical and Computational Physics, University of Hong Kong, Hong Kong, China*

(Dated: February 29, 2016)

[*] E-mail: weisheng.zhao@buaa.edu.cn

[†] E-mail: yanzhou@hku.hk

Supplementary Figures

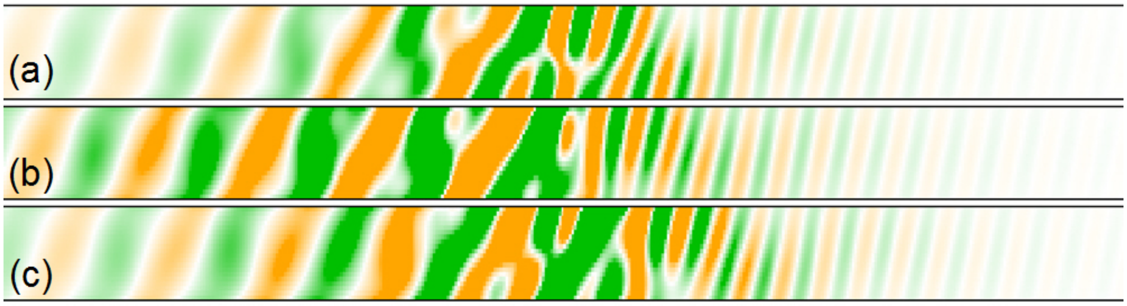

Supplementary Figure 1. Snapshots of the z-component of the magnetization in magnetic strips in the vicinity of the field pulse traveling at a constant speed with different moving field profiles. The external rectangular shape field pulse (**H**) is applied to the 12-μm-long, 100-nm-wide, and 10-nm-thick magnetic strip with a magnitude of 10 mT in the z-direction and constant speed of v_h. Here, $D = 1$ mJ m^{-2} and $v_h = 1100$ m s^{-1}. (**a**) The width in the x-direction and length in the y-direction of the moving magnetic field equal 12 nm and 100 nm, respectively. (**b**) The width in the x-direction and length in the y-direction of the moving magnetic field equal 50 nm and 100 nm, respectively. (**c**) The width in the x-direction and length in the y-direction of the moving magnetic field equal 100 nm and 100 nm, respectively. The color scale representing the out-of-plane component of the magnetization m_z is shown in the Figure 1 of the main text.

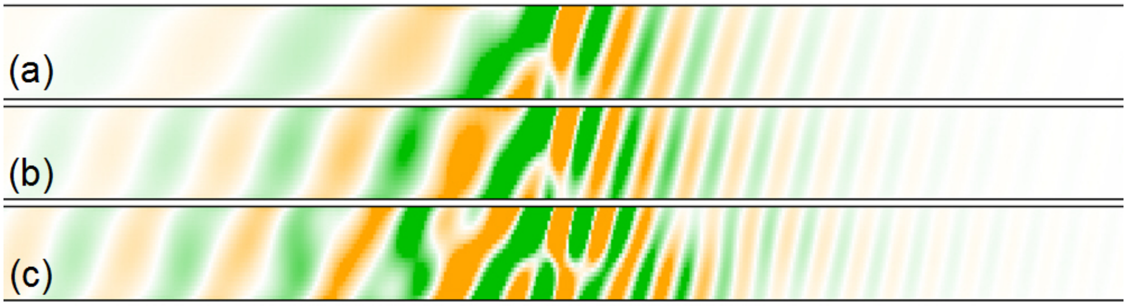

Supplementary Figure 2. Snapshots of the z-component of the magnetization in magnetic strips with different thicknesses in the vicinity of the field pulse traveling at a constant speed. The external rectangular shape field pulse (**H**), 12 nm wide in the x-direction and 100 nm long in the y-direction, is applied to the 12-μm-long, and 100-nm-wide magnetic strip with a magnitude of 10 mT in the z-direction and constant speed of v_h. Here, $D = 1$ mJ m^{-2} and $v_h = 1100$ m s^{-1}. (**a**) The thickness of the magnetic strip equals 1 nm. (**b**) The thickness of the magnetic strip equals 5 nm. (**c**) The thickness of the magnetic strip equals 10 nm. The color scale representing the out-of-plane component of the magnetization m_z is shown in the Figure 1 of the main text.
